# Differential Effects of High-Protein Diets Derived from Soy and Casein on Blood–Brain Barrier Integrity in Wild-type Mice

**DOI:** 10.3389/fnut.2017.00035

**Published:** 2017-07-24

**Authors:** Matthew Snelson, John C. L. Mamo, Virginie Lam, Corey Giles, Ryusuke Takechi

**Affiliations:** ^1^Faculty of Health Sciences, School of Public Health, Curtin University, Bentley, WA, Australia; ^2^Curtin Health Innovation Research Institute, Curtin University, Bentley, WA, Australia

**Keywords:** blood–brain barrier, casein, neuroinflammation, high-protein diet, soy

## Abstract

A number of studies report that a diet high in protein influences cognitive performance, but the results are inconsistent. Studies demonstrated that protein from different food sources has differential effects on cognition. It is increasingly recognized that the integrity of cerebrovascular blood–brain barrier (BBB) is pivotal for central nervous system function. However, to date, no studies have reported the effects of high-protein diets on BBB integrity. Therefore, in this study, the effects of diets enriched in casein or soy protein on BBB permeability were investigated. Immunomicroscopy analyses of cerebral parenchymal immunoglobulin G extravasation indicated significant BBB disruption in the cortex of young adult mice maintained on high-casein diet for 12 weeks, while no signs of BBB dysfunction were observed in mice fed with control or high-soy protein diet. Moreover, cortical expression of glial fibrillary acidic protein (GFAP) was significantly greater in mice fed the high-casein diet compared to control mice, indicating heightened astrocyte activation, whereas mice maintained on a soy-enriched diet showed no increase of GFAP abundance. Plasma concentrations of homocysteine were markedly greater in mice maintained on a high-casein diet in comparison to control mice. Collectively, these findings suggest that a diet enriched in casein but not soy protein may induce astrocyte activation through exaggerated BBB permeability by increased plasma homocysteine. The outcomes indicate the differential effects of protein sources on BBB and neuroinflammation, which may provide an important implication for dietary guidelines for protein supplementation.

## Introduction

The blood–brain barrier (BBB) is a unique feature of the neurovascular unit that physically separates brain from blood. The BBB is comprised of the endothelium, accompanying basal lamina and supporting pericytes and astrocytes. The plasma membranes of the endothelial cells contain adherens and tight junction proteins that limit the paracellular space between endothelial cells to reinforce strict control of transport of substances between blood and brain compartments (1, 2). The BBB has recently garnered significant research interest within the area of neurodegenerative disorders whereby increasing evidence suggests chronic and acute increases in BBB permeability may impose substantial stress on neuronal integrity and function (3). A dysfunctional BBB results in cerebral extravasation of blood-borne neuroactive molecules including pro-inflammatory cytokines and thereafter, potential genesis of reactive oxygen species. Persistently elevated neuroinflammation and oxidative stress can increase endoplasmic reticulum stress, protein misfolding, and DNA and cellular damage, which may eventually result in the loss of neurons (4).

There is increasing evidence that dietary macro- and micronutrients regulate cerebral capillary integrity and function (5–7). Western styled high-fat diets enriched in saturated fats and cholesterol attenuate tight junction protein expression (8, 9), resulting in blood-to-brain extravasation of plasma proteins and macromolecules, neurovascular inflammation and with long-term feeding, cognitive decline. However, equicaloric high-fat diets containing principally mono- or polyunsaturated fatty acids (PUFAs) were found to have no detrimental effects on BBB integrity in preclinical rodent model studies. Carbohydrate-induced models of insulin resistance also indicate aberrations in BBB integrity preceding onset of frank diabetes and cognitive dysfunction.

Surprisingly, putative regulatory effects of dietary protein on the barrier function of cerebral capillaries have not been previously reported. Paradoxical clinical and population cognitive studies are consistent with potential differential effects of dietary protein source on brain capillary integrity. Some findings suggest that older-aged subjects may cognitively benefit from diets enriched in protein (10, 11), while other studies indicate potential detrimental effects (12). Differential effects were indicated by Vercambre et al. who found in a study of 4,809 participants that higher poultry intake was associated with better cognitive function, whereas no significant associations were observed with beef, pork, and lamb (13).

In this study, two commonly consumed protein-rich formulations were considered to investigate potential regulatory effects on brain capillary integrity and function. Casein describes a group of phosphoproteins commonly found in mammalian milk. Being hydrophobic and forming a gelatinous emulsion during digestion, casein supplementation is commonly used for sustained release of amino acids. The alternate source studied was soy protein, which contains principally globulin proteins that are hydrophilic. Milk- and pulse-derived protein isolates both contain a complement of biologically active or metabolic proteins. The findings reported in this study are therefore indicative of synergistic effects of complex food commodity protein concentrates provided in a physiologically relevant context.

## Materials and Methods

### Animals and Intervention

Thirty female wild-type C57BL/6J mice, aged 6 weeks, were purchased from Animal Resource Centre (WA, Australia) and were randomly allocated to one of three groups: control, casein, or soy (*n* = 10 per group). The mice in control group received a modified standard maintenance chow, AIN-93M with calcium and phosphorus content increased to maintain equality with the intervention diets for 12 weeks. Both high-protein diets provided 55% kJ total energy from a protein source (see Table 1 for detail), which is similar to previous studies where Atkins diet was investigated (14). All diets were prepared by Specialty Feeds (WA, Australia). The mice had *ad libitum* access to diet and water and were kept in an accredited animal holding facility. Air pressure, temperature, and lighting (12 h light/dark) were all carefully regulated. All experiments were performed according to the Australian Code of Practice for the Care and Use of Animals for Scientific Purposes. Animal housing and experimental procedures were approved by Animal Ethics Committee (Curtin University approval no. AEC_2011_30A). The female mice were selected to allow group casing that is required by the ethics and also to make the study consistent with our previous studies.

**Table 1:** Dietary composition data sheet (% w/w).

Diet	Control	Casein	Soy
Total fat	4.0	4.0	6.2
Saturated fat	0.3	0.3	0.6
Monounsaturated fat	2.3	2.3	2.8
Polyunsaturated fatty acid (PUFA)	1.4	1.4	2.7
Total n-3 PUFA	0.56	0.56	0.71
Total n-6 PUFA	0.86	0.86	2.00
Total digestible energy from lipids	9.7	9.5	9.2
Total protein	13.6	50.0	50.0
Methionine	0.60	1.57	0.95
Cysteine	0.05	0.15	0.67
Valine	3.24	3.24	2.67
Leucine	1.30	4.64	4.44
Isoleucine	0.60	2.25	2.67
Threonine	0.60	2.05	2.06
Lysine	1.00	3.84	3.50
Phenylalanine	0.70	2.54	2.89
Tyrosine	0.70	2.70	2.11
Tryptophan	0.20	0.70	0.60
Total digestible energy from protein	15.5	55.2	55
Fiber	4.7	4.7	4.7
Calcium	0.80	0.80	0.80
Phosphorus	0.60	0.60	0.60
Digestible energy (MJ/kg)	15	15.6	16

*The control diet was AIN-93M standard rodent chow modified for calcium and phosphorus to ensure equality of these minerals across all diets. Both protein-enriched diets provided approximately 55% kJ from protein with the protein source being casein and soy protein isolate for the casein and soy diets, respectively*.

### Tissue and Plasma Collection

After 12 weeks of dietary intervention, blood samples were collected by cardiac puncture, and plasma was collected and stored at −80°C. Following exsanguination, the brains were removed and washed in phosphate-buffered saline. The brains were hemisectioned, and the right brain hemispheres were fixed in 4% paraformaldehyde for 24 h at 20°C and thereafter, underwent cryoprotection in 20% sucrose solution for 72 h at 4°C. Subsequently, the samples were frozen in isopentane/dry ice and stored at −80°C.

### Plasma Homocysteine Analysis

Total homocysteine was assayed in plasma with a chemiluminescent microparticle immunoassay (Abbott Diagnostics, IL, USA). Plasma was diluted 1:10 or 1:5 with Multi-Assay Manual Diluent No. 7D82-50 (Abbott Diagnostics) prior to analysis by Architect i2000SR Analyzer (Abbott Diagnostics) at PathWest (WA, Australia).

### Quantitative 3-D Immunofluorescent Microscopy Analyses for BBB Permeability and Astrocyte Activation

The measurement of cerebral parenchymal extravasation of endogenous immunoglobulin g (IgG) by using 3-D immunofluorescent confocal microscopy has been widely utilized as a surrogate marker for BBB permeability and established within our laboratory as described previously (7, 15). Briefly, 20 µm cryosections were prepared from the right hemispheres. After blocking of non-specific binding sites, polyclonal goat anti-mouse IgG antibody conjugated with Alexa 488 (Life Technologies, MA, USA) was applied to the sections for 20 h at 4°C. Blood vessels were identified by the IgG staining and nuclei staining (DAPI). Parenchymal extravasation of IgG outside of those identified vessels was selected by an automated threshold-based measurement module of Volocity 3-D image analysis software that were subsequently confirmed and adjusted manually by the investigators.

Glial fibrillary acidic protein (GFAP), a marker of astrogliosis and astrocytosis, was used as a surrogate indicator of neuronal insults including neuroinflammation, as previously described (7). Briefly, sections were incubated with polyclonal rabbit anti-mouse GFAP (Abcam, CB, UK) at 4°C for 20 h. The tissues were then incubated with goat anti-rabbit IgG conjugated with Alexa 488 for 2 h at 20°C. The slides were counterstained with DAPI and mounted with anti-fading medium.

3-D immunofluorescent images were captured for quantitation by an established methodology (8, 15, 16). Briefly, the images were captured with ApoTome optical sectioning system and MRm digital camera connected to AxioVert 200M (Carl Zeiss, Germany). Images were taken at ×200 magnification (430 µm × 322 µm), with each 3-D image consisting of 12 Z-stack images with the distance between the Z stack slices being 1.225 µm, optimized by Nyquist theory. To minimize the bias, the images were taken randomly in both the hippocampal and cortical brain regions. For each mouse killed, approximately 20 images were taken from the cortex, and approximately 6 images were taken from the hippocampus, which covered more than half of each brain region. For quantification of IgG and GFAP cerebral abundance, Volocity 3-D image analysis software (PerkinElmer, UK) was utilized to calculate voxel intensity of the fluorescent dye of interest, expressed per volume unit.

### Statistical Analysis

All data were entered and calculated on Excel (Microsoft, CA, USA) and expressed as mean ± SEM. Data analysis was completed using Prism 7 (GraphPad, CA, USA). D’Agostino and Shapiro–Wilk normality tests were used to assess the Gaussian distribution. For the data that were normally distributed, one-way ANOVA with Fisher’s LSD *post hoc* test was used. For the data that were not normally distributed, non-parametric Kruskal–Wallis test with Mann–Whitney *post hoc* analysis was used. Statistical significance was noted at *p* < 0.05. Associations were analyzed with Spearman’s correlation coefficient.

## Results

### High-Protein Diets Were Well Tolerated

Modified AIN-93M diet and protein-enriched diets were well tolerated by mice with similar rates of growth for casein and soy groups of mice compared to the mice maintained on control chow containing 15.5% (kJ) protein; no adverse effects were observed. Following 12 weeks of dietary intervention, the weight of mice in the treatment groups was not significantly different from each other group (control: 21.3 ± 0.44 g vs casein: 19.8 ± 0.47 g vs soy: 19.4 ± 0.33 g).

### BBB Integrity Is Compromised by a Chronic Ingestion of Diet High in Casein but Not in Soy

**Figure 1:**
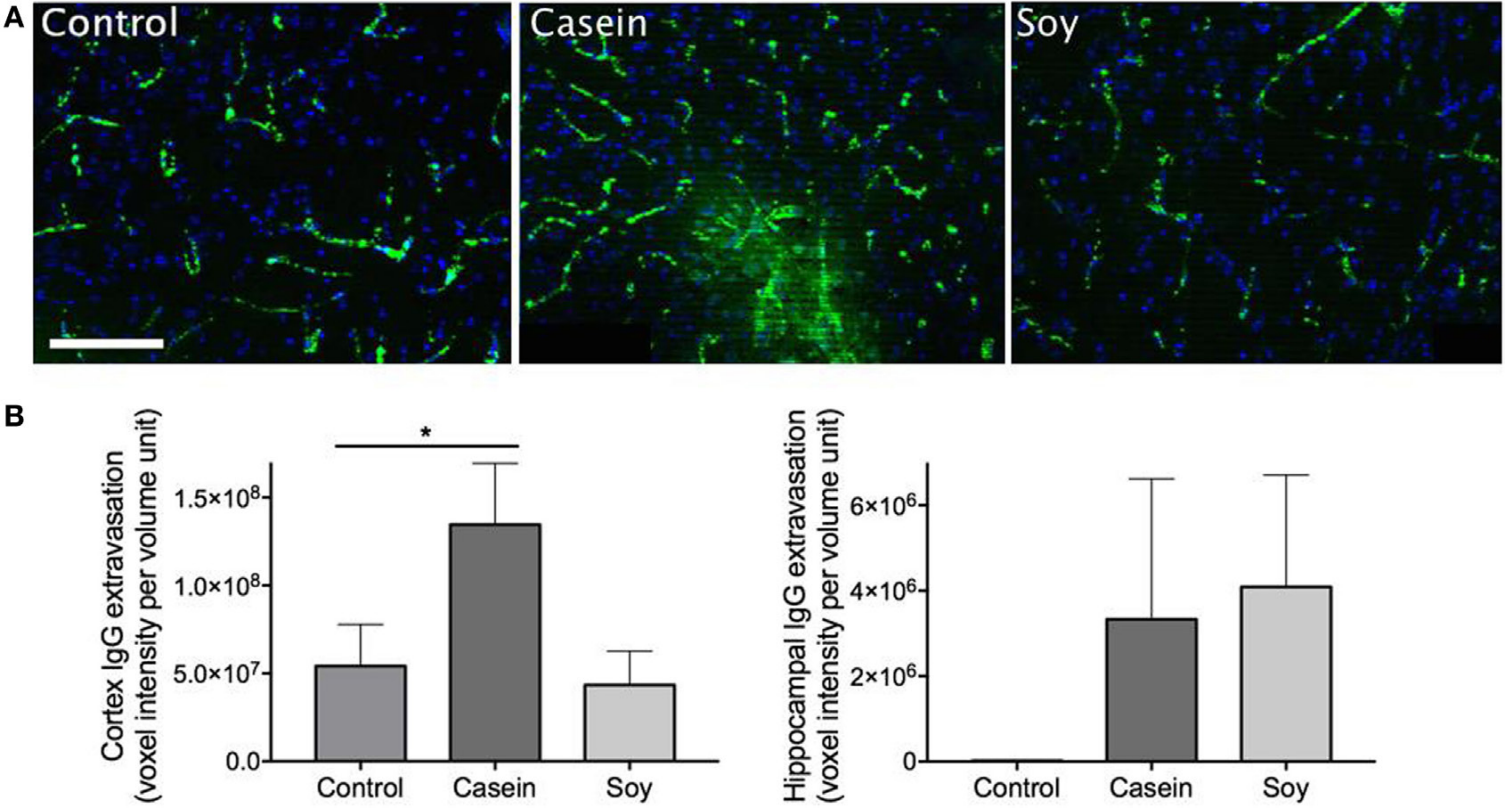
Semiquantitative analyses of cerebral parenchymal immunoglobulin g (IgG) extravasation. Cerebral capillary integrity was assessed *via* immunofluorescent detection of the plasma protein IgG within the cerebral parenchyma in mice maintained on control chow, high-casein diet, or high-soy protein diet. **(A)** The micrographs show the representative images of peri-vascular IgG (green) in cortex region with DAPI counterstaining of cell nuclei (blue). Scale bar = 100 µm. **(B)** Semiquantitative analysis of IgG extravasation in cerebral cortex and hippocampal formation is shown. The statistical significance is indicated with * at *p* < 0.05 (Mann–Whitney test, *n* = 10). Data shown as mean ± SEM.

The effect of high-protein diets on parenchymal IgG extravasation is presented in Figure 1. Substantial peri-vascular parenchymal extravasation of plasma-derived IgG was observed in the mice maintained on a diet enriched in casein for 12 weeks, indicating substantial breakdown of BBB, while no distinct evidence of BBB disruption was found in control and soy protein-fed mice (Figure 1A). The semiquantitative analyses of 3-D immunofluorescent microscopy images revealed that mice fed with high-casein diet showed significantly elevated IgG extravasation in the cerebral cortex region (Figure 1B). The cortical IgG extravasation in mice fed with high-soy diet was comparable to control mice. The IgG extravasation in the hippocampal formation of high-casein- and -soy protein-fed mice was not significantly different from control mice, although it showed some increasing trend (Figure 1B).

### High-Casein Diet Induces Astrocyte Activation

**Figure 2:**
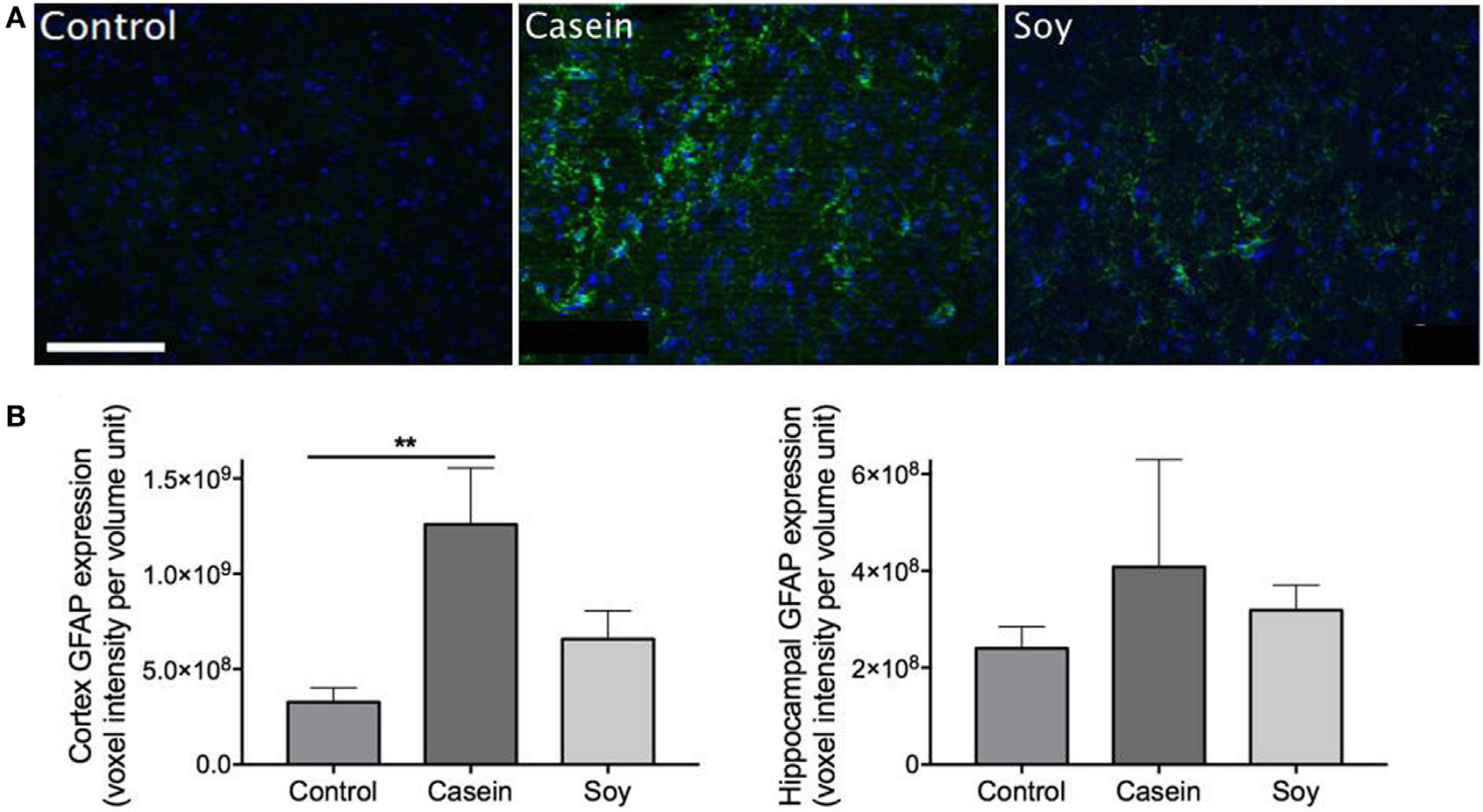
Semiquantitative analyses of cerebral glial fibrillary acidic protein (GFAP) expression. Neuroinflammation was assessed *via* immunofluorescent detection of GFAP expression within the cerebral parenchyma of mice maintained on control chow, high-casein diet, or high-soy protein diet. **(A)** The micrographs show representative immunofluorescent staining of GFAP (green) with DAPI counterstaining (blue) in the cortex. Scale bar = 100 µm. **(B)** Semiquantitative analysis of GFAP extravasation in cerebral cortex and hippocampal formation are shown. The statistical significance is indicated with ** at *p* < 0.01 (Mann–Whitney test, *n* = 10). Data shown as mean ± SEM.

Cerebral parenchymal GFAP expression was markedly elevated in the mice maintained on high-casein diet for 12 weeks indicating heightened neuronal insults, while exaggerated astrogliosis and astrocytosis were not observed in soy protein-fed mice (Figure 2A). Semiquantitative microscopy analyses showed that GFAP expression in the cortex of mice fed with high-casein diet was significantly higher than the control mice, while no significant increase was observed in soy protein-fed mice (Figure 2B). No significant changes of GFAP expression were observed in hippocampal region of mice maintained on high-protein diets.

### Plasma Homocysteine Was Elevated in High-Casein-Fed Mice and Was Positively Associated with BBB Dysfunction

**Figure 3:**
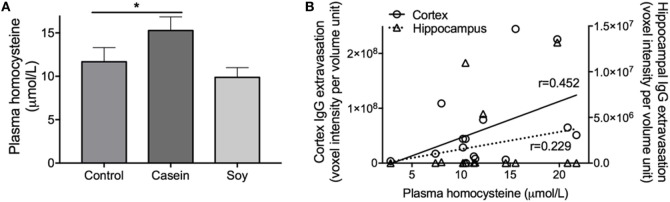
Plasma concentration of homocysteine. **(A)** Plasma homocysteine concentration was determined in mice maintained on control chow, high-casein diet, or high-soy protein diet using an immunoabsorbent assay. The statistical significance is indicated with * at *p* < 0.05 (one-way ANOVA with Fisher’s LSD *post hoc* test, *n* = 10). Data shown as mean ± SEM. **(B)** The associations between plasma homocysteine levels and cerebral parenchymal immunoglobulin g (IgG) extravasation were determined by Pearson’s correlation coefficient analysis.

Plasma concentration of homocysteine showed 30% increase in the mice fed with high-casein diet for 12 weeks compared to control mice (Figure 3A). In mice maintained on high-soy diet, plasma homocysteine remained similar to control mice. The correlation analysis showed that the plasma levels of homocysteine were moderately associated with cortex (*r* = 0.452) and hippocampal (*r* = 0.229) IgG extravasation (Figure 3B).

## Discussion

Previous studies report that differential effects of protein derived from various sources may have different effects on cognitive function. While emerging evidence suggests that BBB integrity may be central to neurocognitive performance, no studies to date examined the effects of high-protein consumption on BBB permeability. Therefore, this study aimed to investigate the effects of high-protein diet derived from casein or soy protein on the integrity of BBB.

The abundance of brain parenchymal IgG is a commonly used marker of compromised BBB integrity (17, 18). Our results demonstrated that chronic ingestion of diet enriched in casein induced substantial BBB disruption in the cortex region resulting in non-specific blood-to-brain extravasation of plasma-derived proteins, while a diet enriched in soy protein showed no significant effects on BBB permeability. Concomitantly, significant elevation of astrogliosis and astrocytosis assessed by GFAP expression was only observed in the cerebral cortex of mice maintained on a diet enriched in casein. Consistent with our findings, there is considerable evidence that supports a causal relationship between exaggerated neuroinflammation and dysfunctional BBB (19–21); a loss of cerebral capillary integrity has been shown to be associated with both peripheral inflammation (22–24) and more recently, cerebral inflammation (25). These data collectively suggest that a high-protein diet derived from casein but not soy protein may induce BBB disruption and thereafter induce neuroinflammation.

Given that cortical BBB dysfunction and neurovascular insults including neuroinflammation were associated with the casein-enriched diet but not with the high-soy diet, it is plausible to suggest that it is not the overall protein content *per se*, but rather the other components within the casein diet that may exert deleterious effects on the BBB. Studies report that homocysteine is associated with compromised cerebrovascular function including BBB dysfunction (26). Tyagi et al. demonstrated that hyperhomocysteinemia induces structural and functional alterations of BBB by promoting oxidative stress (27). Moreover, studies have also shown that homocysteine increases BBB permeability and neuroinflammation in mouse models of cognitive dysfunction (28, 29). In alignment with previous findings, we demonstrated that casein diet-induced BBB dysfunction was accompanied with a significant increase in plasma homocysteine. Furthermore, correlation analysis showed a positive association between plasma homocysteine levels and BBB permeability. It has been shown previously that certain amino acids found in protein-rich diets, specifically the amino acid methionine, are associated with elevated plasma homocysteine levels (30, 31). In this study, the methionine content of casein diet was significantly higher than the control and soy protein diet [casein: 1.57% (w/w) vs control: 0.60% vs soy: 0.95%]. Thus, these data suggest that ingestion of high-casein diet may induce BBB dysfunction through its high methionine content increasing plasma homocysteine concentration.

Alternatively, it is possible the differential effects induced by casein/soy-enriched diets may be due to specific component/s of the soy diet that may attenuate detrimental effects of a high-protein diet on BBB integrity. Cysteine has been shown to attenuate methionine-induced hyperhomocysteinemia (32). Indeed, the soy-enriched diet utilized in this study had a fourfold higher concentration of cysteine compared to the casein diet, which may plausibly attenuate the proposed effect of a high protein-induced BBB disruption. In addition, the high-soy protein diet contained approximately twofold greater content of PUFA. Our laboratory has previously demonstrated the differential effects of fatty acids on the BBB integrity (8). Consistent with the findings of the current study, we showed that diets enriched in PUFA exerted BBB protective effects, while saturated fats were shown to significantly deteriorate BBB function. In addition to these BBB protective nutrients in soy protein products, other nutrients that are not specifically measured in the current study may contribute to the beneficial effects of soy protein diet, i.e., isoflavones (33).

To the authors’ best knowledge, this is the first study to report the effects of high-protein diets on measures of cerebral capillary BBB integrity and neuroinflammation. In summary, despite the provision of equivalent kilojoules derived from both casein and soy protein diets (55% kJ), a casein–protein-enriched diet was associated with increased probability of BBB disruption and neuroinflammation in the cerebral cortex, while these effects were not observed in a soy-based diet. The detrimental effects of casein diet may be attributed to the high methionine content, possibly inducing hyperhomocysteinemia and thereafter, downstream oxidative pathways. The outcomes of this study may provide further insight on the underlying mechanisms of high-protein induced cognitive decline, and may provide important considerations for dietary guidelines of protein supplement.

## Ethics Statement

Animal housing and experimental procedures were approved by Animal Ethics Committee (Curtin University approval no. AEC_2011_30A).

## Author Contributions

The study was designed by MS, JM, and RT; samples were collected and analyzed and data interpretation and manuscript preparation were undertaken by MS, JM, VL, CG, and RT. All the authors approved the final version of the paper.

## Conflict of Interest Statement

The authors declare that the research was conducted in the absence of any commercial or financial relationships that could be construed as a potential conflict of interest.
